# Dissolution Improvement of Atorvastatin Calcium using Modified Locust Bean Gum by the Solid Dispersion Technique

**DOI:** 10.3797/scipharm.1301-23

**Published:** 2013-12-09

**Authors:** Dharmila Panghal, Manju Nagpal, Gurjeet Singh Thakur, Sandeep Arora

**Affiliations:** Chitkara College of Pharmacy, Chitkara University, Chandigarh-Patiala National Highway, Rajpura-140401, Punjab, India.

**Keywords:** Modified locust bean gum, Solvent evaporation, Solid dispersion, Co-grinding mixture, Content uniformity

## Abstract

The present research was aimed at the enhancement of the dissolution rate of atorvastatin calcium by the solid dispersion technique using modified locust bean gum. Solid dispersions (SD) using modified locust bean gum were prepared by the modified solvent evaporation method. Other mixtures were also prepared by physical mixing, co-grinding, and the kneading method. The locust bean gum was subjected to heat for modification. The prepared solid dispersions and other mixtures were evaluated for equilibrium solubility studies, content uniformity, FTIR, DSC, XRD, *in vitro* drug release, and *in vivo* pharmacodynamic studies. The equilibrium solubility was enhanced in the solid dispersions (in a drug:polymer ratio of 1:6) and other mixtures such as the co-grinding mixture (CGM) and kneading mixture (KM). Maximum dissolution rate was observed in the solid dispersion batch SD3 (i.e. 50% within 15 min) with maximum drug release after 2 h (80%) out of all solid dispersions. The co-grinding mixture also exhibited a significant enhancement in the dissolution rate among the other mixtures. FTIR studies revealed the absence of drug-polymer interaction in the solid dispersions. Minor shifts in the endothermic peaks of the DSC thermograms of SD3 and CGM indicated slight changes in drug crystallinity. XRD studies further confirmed the results of DSC and FTIR. Topological changes were observed in SEM images of SD3 and CGM. *In vivo* pharmacodynamic studies indicated an improved efficacy of the optimized batch SD3 as compared to the pure drug at a dose of 3 mg/kg/day. Modified locust bean gum can be a promising carrier for solubility enhancement of poorly water-soluble drugs. The lower viscosity and wetting ability of MLBG, reduction in particle size, and decreased crystallinity of the drug are responsible for the dissolution enhancement of atorvastatin. The co-grinding mixture can be a good alternative to solid dispersions prepared by modified solvent evaporation due to its ease of preparation and significant improvement in dissolution characteristics.

## Introduction

Advancements in molecular screening methods for potential drug molecules led to the identification of an increasing number of poorly water-soluble drugs. About 40% of the new chemical entities that have been discovered are poorly water-soluble. Poor aqueous solubility leads to decreased *in vivo* efficacy of the drugs, thereby resulting in low bioavailability. Various approaches to increase the solubility are the use of surfactants, complexation with cyclodextrins, polymorphism, salt formation, micronization, and emulsification. However, there are substantial limitations with each of these techniques. Solid dispersions (SD) have created considerable interest as the potential means of improving the dissolution rate and hence the bioavailability of poorly water-soluble drugs. Moreover, the formulation of drugs as solid dispersions offers a variety of processing and excipient options that allow for flexibility while formulating oral delivery systems for poorly water-soluble drugs [1, 2]. The concept of solid dispersions was introduced by Sekiguchi and Obi in the1960s as the dispersion of one or more active ingredients in an inert carrier matrix at solid state [3]. Drug particles in SD need not necessarily exist in the micronized state. A fraction of the drug might molecularly disperse in the matrix, thereby forming a solid dispersion. When the solid dispersion is exposed to aqueous media, the carrier dissolves and the drug released as fine colloidal particles [4]. The use of natural polymers as alternative carriers has generated a great interest in novel drug delivery, particularly for industrial applications as well as to reduce production costs and toxic effects. But high viscosity and pulverization are important drawbacks with these polymers [5]. Therefore, studies using modified forms of the natural polymers such as locust bean gum (LBG), guar gum, and gum karaya (desired swelling and reduced viscosity characteristics) have been reported for preparing solid dispersions of hydrophobic drugs [5–7]. LBG is a natural polymer, extracted from the seeds (kernels) of the carob tree Ceratonia siliqua (Family - Leguminosae or Fabaceae) and also known as carob bean gum or carubin. It is used as a thickening and stabilizing agent. LBG is used widely due to its high swelling and water retention capacity, binding capacity, and chemical compatibility [8, 9]. Atorvastatin is a member of the statins category of drugs, and is used for lowering blood cholesterol levels. It has a short half-life (T_max_, 1–2 h) and good intestinal permeability. The low oral bioavailability of the drug (12%) is due to its low aqueous solubility (0.1 mg/mL), crystalline nature, and hepatic first-pass metabolism [10]. Administration in higher doses possibly leads to various toxic effects such as liver abnormalities, arthralgia, and kidney failure etc. [11]. Solubility enhancement of atorvastatin has been revealed through various experimental evidences such as salt formation [12] and inclusion complexes with β-cyclodextrin [13]. The solid dispersion of atorvastatin has been demonstrated using mannitol, PEG-4000 and PVP-K30 [14]. The current study investigates the impact of modified locust bean gum (MLBG) on the solubility enhancement of the poorly water-soluble drug, atorvastatin calcium. The effect of concentration of the polymer and the method of preparation on solubility enhancement were explored. Equilibrium solubility, content uniformity, *in vitro* dissolution studies, infrared spectroscopy, Scanning Electron Microscopy (SEM), Differential Scanning Calorimetry (DSC), and X-ray diffraction (XRD) studies were carried out to explain the phenomenon.

## Results and Discussion

### Characterization of LBG and MLBG

Various physical parameters which were evaluated for LBG and MLBG are depicted in Table 1. No chemical changes occurred during heating at high temperatures, rather changes in viscosity were observed. The results revealed a decrease in viscosity (one-third) of MLBG with almost the same swelling index and hydration capacity as compared to LBG. The changes in volatile acid content may be responsible for the decrease in viscosity [7]. The purpose of the modification of natural polymers was to reduce the viscosity of the polymer while retaining its swelling capacity, which made them useful for solubility enhancement purposes and also they can be used in higher ratios. Also, the polymers (LBG and MLBG) exihibited good flow properties as indicated by Carr’s index values (CI) and angle of repose, which further helps in formulation development. No change in density values was observed in MLBG.

### Evaluation of Solid Dispersions

#### Equilibrium Solubility Studies

Equilibrium solubility data of various mixtures are depicted in Table 2. The increase in solubility was observed in the solid dispersions and other mixtures (KM and CGM) which is more than that of the pure drug. This may be due to the wetting ability of MLBG, and reduced particle size during trituration. However, MLBG added in higher concentrations led to a decrease in the solubility of the solid dispersions (SD4 and SD5). The increased viscosity of the mixture may be responsible for this. No increase in solubility was observed in the physical mixture.

#### Content Uniformity

Content uniformity data of the solid dispersion batches (SD1-SD5), various mixtures, and marketed formulation (Lipitor) are shown in Table 2. Content uniformity was observed in the range of 92.6 ± 0.09 to 100.4 ± 0.01, indicating uniform distributions or dispersions of the drug in various mixtures.

#### Fourier Transform Infrared Spectrum Studies (FTIR)

**Fig. 1:**
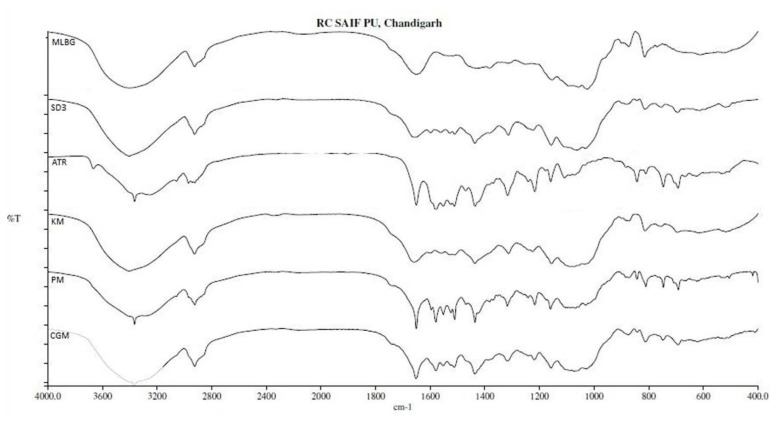
Overlay diagram of FTIR spectra of MLBG, SD3, pure drug atorvastatin calcium (ATR), KM, PM, and CGM (from top to bottom)

FTIR spectra of samples were recorded using FTIR as to ascertain the presence of different functional groups. The FTIR spectra of the pure drug, MLBG, and solid dispersion SD3 and other mixtures (PM, KM, and CGM) are shown in Figure 1. The FTIR spectrum of the pure drug showed the characteristic peaks at 1650.76 due to C=O stretching, at 3364.90 due to O-H stretching, 3252.43 due to N-H stretch, 1435.82 due to C-F stretching, 1316.56 due to C-O stretching, 1217.08 due to C-N stretching, and at 692.62 due to aromatic out-of-plane bend. The FTIR spectrum of MLBG showed C-H stretching at 2925.54, CH_2_ bend at 1432.10, and C-O stretching at 1024.81cm^−1^. Almost all the characteristic peaks of the drug (1435.82, 1316.56, 1217.08, and 690.62) were observed (with minor shifts) in the FTIR spectra of the solid dispersion SD3 and various mixtures. The presence of characteristic peaks of the drug in the solid dispersion and other mixtures indicated the absence of any chemical interaction of the drug with the polymer MLBG. However, the disappearance of the peak at 3252.43 (N-H stretch) may be due to overlapping of the drug particles by the presence of excess polymer.

#### Differential Scanning Calorimetry (DSC)

**Fig. 2:**
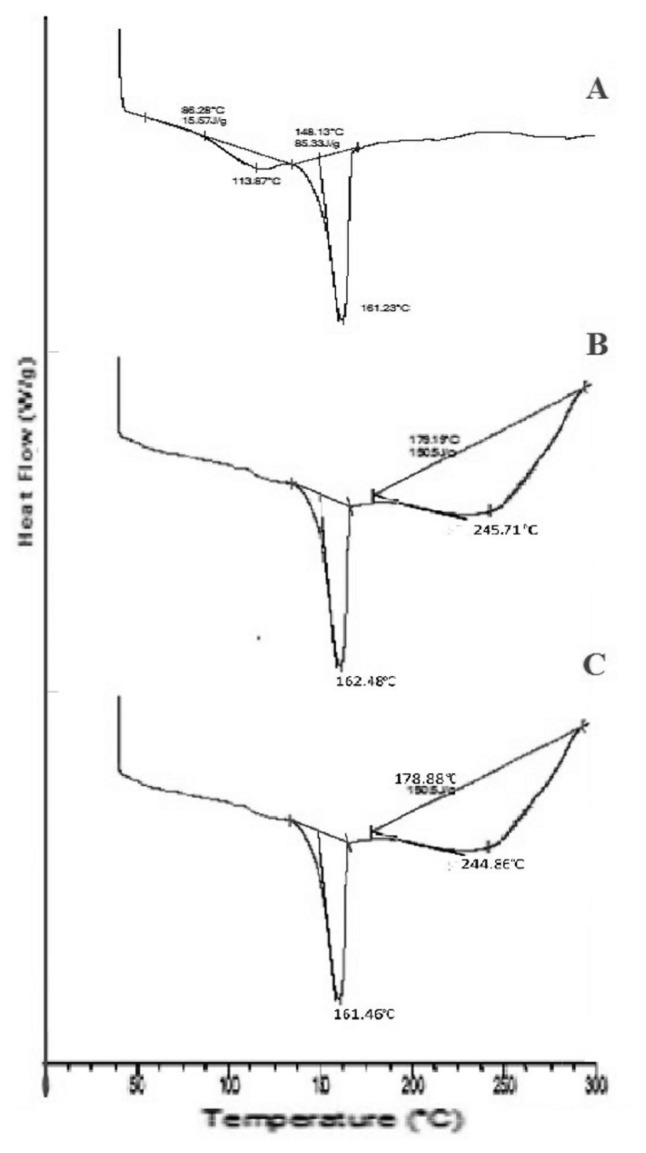
Overlay diagram of DSC of A) pure drug; B) SD3; and C) CGM

The DSC thermogram of atorvastatin calcium (Figure 2) showed a sharp endothermic peak at 161.23°C with an enthalapy of fusion 85.33 J/g corresponding to its melting point, which indicates its crystalline nature. The DSC studies did not reveal a significant change in the crystalline state of the drug. The presence of an endothermic peak with a slight shift and decreased intensity (at 162.48°C in SD3 and at 161.46°C in CGM) in the DSC thermogram of the various mixtures revealed drug integrity when mixed with MLBG. These minor deviations indicated a slight conversion of the drug from its crystalline to amorphous state.

#### Scanning Electron Microscopy (SEM)

**Fig. 3:**
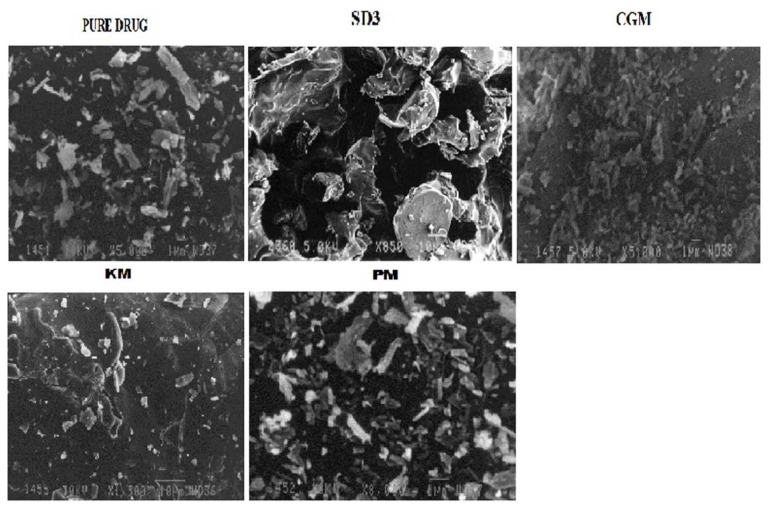
Scanning Electron Micrographs of a) Pure drug; b) solid dispersion formulation SD3; c) Co-grinding Mixture, CGM; d) Kneading Mixture, KM; e) Physical Mixture, PM

Scanning electron micrographs of the drug, solid dispersion SD3, KM, CGM, and PM are shown in Figure 3. The drug (atorvastatin calcium) (Figure 3a) appeared as a smooth-surfaced rectangular crystalline structure and the same was observed in the kneading mixture and physical mixture (Figure 3d and 3e). However, topological changes were observed in solid dispersion SD3 and CGM (Figure 3b and 3c). This may be due to the wetting ability of MLBG, synergistic effect of trituration, and solubilization by the solvent.

#### X-Ray Diffraction (XRD)

**Fig. 5:**
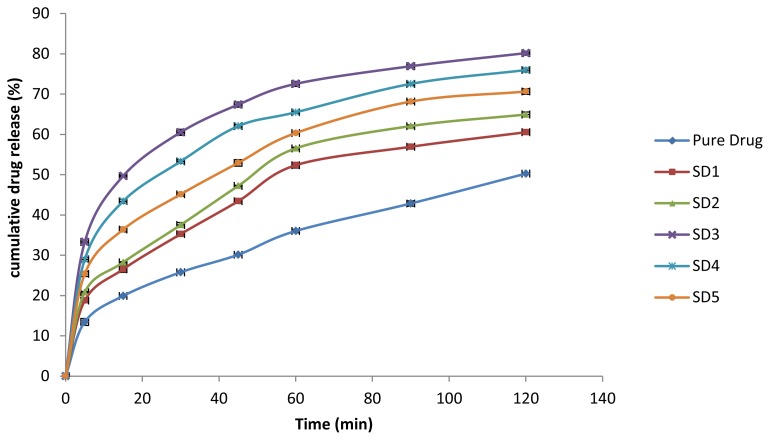
*In vitro* release profile of various solid dispersions (SD1–SD5) and pure drug

The presence or absence of crystallinity of the drug and polymer was determined by the X-Ray Diffraction studies by comparing some representative peak heights in the diffraction of the solid dispersions with that of the pure drug. The XRD pattern of the drug, MLBG, solid dispersion SD3, and CGM are shown in Figure 5. Atorvastatin calcium showed sharp peaks of the diffraction angle of 2θ at 8.9716, 10.0738, 12.0574, 16.8777, 19.2928, 21.4610, and 23.1231 with peak intensities of 54.25, 32.95, 28.79, 95.86, 59.25, 100.00, and 46.33 and the areas of 67.90, 54.99, 24.03, 199.96, 98.88, 166.88, and 57.99, respectively. Diffraction patterns of solid dispersion batch SD3 showed broader peaks of the diffraction angle of 2θ at 9.1192, 16.8950, 19.3360, and 21.4785 with peak intensities of 29.47, 100, 59.23, and 50.34 and the decreased areas of 19.92, 33.80, 20.02, and 34.03. Absence of some characteristic peaks of the drug in solid dispersion SD3 and the co-grinding mixture along with the decreased area of some characteristic peaks indicated a partial conversion of the crystalline form of the drug to amorphous form (Table 3).

#### In vitro Drug Release

**Fig. 6:**
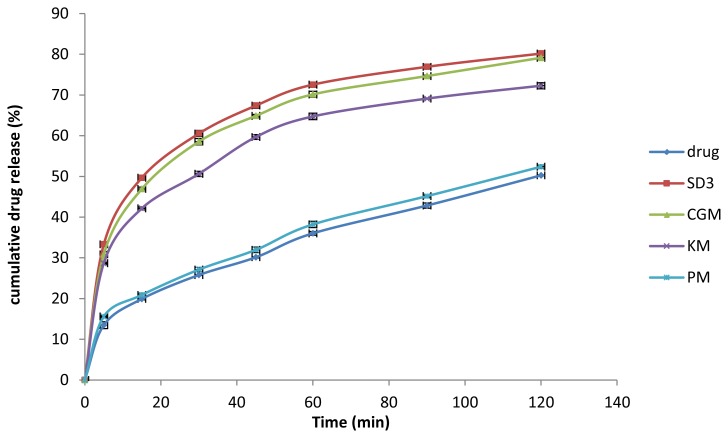
Comparative *in vitro* release profiles of SD3, KM, CGM, PM, and pure drug

The comparative *in vitro* drug release profile of the various solid dispersions (SD1-SD4) and the pure drug is shown in Figure 5. All solid dispersions showed an increase in rate and extent of dissolution as compared to the pure drug. The maximum rate (in the first 15 min) and extent of dissolution at the end of 2 h (50% and 80%, respectively) was observed in the solid dispersion SD3 (p< 0.05) out of all the solid dispersions. The addition of the polymer led to enhanced dissolution, but higher ratios of polymer (more than 1:6) decreased the release characteristics as there may be formation of viscous plugs at these concentrations. The drug release behavior also confirmed the equilibrium solubility study results. SD3 was observed as a promising batch and the *in vitro* drug release from SD3, the drug, PM, KM, and CGM was further compared (Figure 6). A significant increase in the dissolution characteristics of CGM and KM was observed as compared to the pure drug (p< 0.05). However, no significant increase in dissolution was observed in PM. CGM showed equivalent drug release characteristics (46.92% in 15 min and 79.13% in 2 h) as that of SD3 which suggests the possibility of CGM as a good alternate for solubility enhancement. Moreover, the easy preparation of CGM as compared to solid dispersions may prove it a suitable alternative to solid dispersions.

#### In vivo Pharmacodynamics Studies

Solid dispersion SD3 was compared with the pure drug during *in vivo* studies. The results are depicted in Table 4. A significant decrease in total cholesterol, LDL, and triglyceride levels was observed in the disease control group when treated with solid dispersion SD3. Two dose levels (1 mg/kg/day and 3 mg/kg/day) were employed and found there was a significant difference between the drug and SD3 group at a higher dose of 3 mg/kg/day as compared to 1 mg/kg/day. The efficacy of the pure drug and SD3 was also compared with the control group. The results indicated better performance of the solid dispersion SD3 (3 mg/kg/day) formulation as compared to the standard pure drug (3 mg/kg/day).

## Conclusion

The present study explored the solubility enhancement of atorvastatin calcium using a modified form of locust bean gum as a carrier. The improvement in the equilibrium solubility of the drug in solid dispersions leads to dissolution enhancement. The synergistic effect of wetting ability of MLBG, decreased particle size of the drug during formulation of mixtures, and decreased crystallinity of the drug may lead to enhanced solubility of the drug. Moreover, the less viscous MLBG produced promising results as it can be used in a higher ratio as compared to unmodified polymer (LBG), in solid dispersion technique. However, the co-grinding method (among other methods) can be a suitable alternate to the solid dispersion technique as it also led to significant improvements in dissolution characteristics as well as its ease of preparation.

## Experimental

### Materials

Atorvastatin was kindly gifted by Abott Healthcare Ltd., Baddi, India and locust bean gum was obtained as a gift sample from Taiyo Lucid Pvt. Ltd., Mumbai, India. All other materials used were of analytical grade.

### Methods

#### Modification of Locust Bean Gum (LBG)

Modified locust bean gum (MLBG) was prepared by the method (with slight modification) reported by Murali Mohan Babu et al [7]. Locust bean gum was placed in a porcelain dish and heated on a sand bath for a period of 30–45 min. The temperature of the sand bath was kept in the range of 80–100°C. The polymer was stirred continuously with a spatula in order to avoid the charring of the polymer. The temperature and time of heating was selected on the basis evaluation of visual changes and viscosity changes in MLBG. The polymer LBG should not get charred when kept at these conditions. The MLBG sample was checked for viscosity changes during the modification. The temperature and time of heating was selected after which no change in viscosity was observed. The prepared modified locust bean gum (MLBG) was sieved through the 80 mesh and kept in a desiccator for further evaluation.

### Characterization of LBG and MLBG

LBG and MLBG were characterized for their swelling behavior, viscosity, hydration capacity, flow properties (angle of repose, tapped density, bulk density, and compressibility index) by the methods already reported [5].

#### Preparation of Solid Dispersions

Solid dispersions were prepared by the modified solvent evaporation method. The drug, atorvastatin calcium (250 mg) was dispersed in 70% v/v ethanol (25 ml). The polymer (MLBG) was dispersed in sufficient water (depending on its wetting ability) separately. The drug solution was poured in a polymer suspension. The drug polymer mixture was evaporated under reduced pressure using a rotary evaporator at 60°C. Various SD formulations (SD1, SD2, SD3, SD4, and SD5) in different ratios (1:2, 1:4, 1:6, 1:8, and 1:10) were prepared. The resultant solid dispersions were sieved through 80 # mesh and stored in a desiccator till further evaluation.

#### Physical Mixing Method

Accurately weighed atorvastatin (100 mg) and modified locust bean gum (600 mg) were mixed thoroughly with the help of a spatula. The resulting physical mixture (PM) was sieved through 80 # mesh and stored in a desiccator till further evaluation.

#### Co-grinding Method

The drug atorvastatin (100 mg) and modified locust bean gum (600 mg) were accurately weighed. The drug and polymer were placed in the mortar and the mixture was ground properly. The resulting co-grinding mixture (CGM) was passed through the 80 # mesh sieve and stored in a desiccator till further evaluation.

#### Kneading Method

The drug and polymer were placed in the mortar and the mixture was kneaded with a small quantity of the ethanol. The kneaded mixture was placed in the hot air oven until the constant weight of the mixture was obtained. After complete drying (evaporation of the solvent), the resulting kneading mixture (KM) was collected and passed through the 80 # mesh sieve and stored in a desiccator till further evaluation.

Table 5 shows the composition of various solid dispersions (SD1-SD5), PM, CGM, and KM.

### Evaluation of Solid Dispersions

#### Equilibrium Solubility Studies

The equilibrium solubility of the pure drug, solid dispersions (SD1–SD5), PM, KM, and CGM was determined in a phosphate buffer (pH 6.8) at 37°C. An equivalent of 10 mg of the drug was added to 50 ml of buffer in conical flasks. The flasks were kept in a shaking incubator for 24 h at 37±0.5°C and further equilibrated in an incubator at 37±0.5°C for 12 h. Then, the solution was filtered using a 0.45 μ Millipore filter and assayed spectrophotometrically at 240 nm.

#### Content Uniformity

An accurately weighed amount of the solid dispersions (SD1-SD5), PM, CGM, KM (equivalent to 5 mg), and the marketed product (Lipitor) were dissolved in 10 ml methanol in a 100 ml volumetric flask and volume was made up to the mark using a phosphate buffer, pH 6.8. The solution was filtered using a 0.45 μ Millipore filter and analyzed for drug content at 240 nm using a UV spectrophotometer.

#### Fourier Transform Infrared Spectroscopy (FTIR)

Infrared absorption spectra of the pure drug, LBG, MLBG, solid dispersions (SD1–SD4), and other mixtures (CGM, KM, and PM) were obtained using the potassium bromide method, under static air using an FTIR spectrophotometer (Perkin Elmer Spectrum 400). About 10 mg of the sample was mixed with dried potassium bromide of equal weight. Pellets were formed by compressing the mixture by using a hydraulic press. Transparent pellets formed in this way were scanned. The spectra were scanned over a frequency range of 4000–00 cm^−1^.

#### Differential Scanning Calorimetry (DSC)

Thermal properties of the pure drug, optimized solid dispersion SD3, and CGM were analysed by Differential Scanning Calorimetry (DSC 821^e^ Mettler Toledo, USA). The sample was sealed in an aluminium pan and scanned from 30 to 300°C at a heating rate of 10°C/min in nitrogen atmosphere.

#### Scanning Electron Microscopy (SEM)

Samples of the pure drug, optimized solid dispersion SD3, PM, KM, and CGM were mounted onto the stubs using double-sided adhesive tape and then coated with a gold palladium alloy (150–200 Å) using a fine coat ion sputter (JEOL JSM-6100). The samples were subsequently analyzed under the scanning electron microscope for external morphology.

#### X-Ray Diffraction (XRD)

Powder X-Ray diffraction patterns were traced employing the X-ray diffractometer (X’pert PRO, PAN analytical, Netherlands) for the samples using Ni-filtered Cu (K-α) radiations, a voltage of 45 kV, and current of 40 mA. The samples were analyzed over the 2θ range of 0–50° with a scan step size of 0.0170° (2θ) and scan step time 25 s.

#### In vitro Drug Release

*In vitro* dissolution was carried out in 900 ml of a phosphate buffer (pH 6.8) at 37±0.5°C with the rotation speed of 50 rpm using a USP dissolution apparatus (Type II). A 5 ml aliquot of dissolution medium was withdrawn at specified intervals (5, 15, 30, 45, 60, 90, and 120 min) and filtered using a 0.45 μ Millipore filter. The samples were suitably diluted and assayed spectrophotometrically at 240 nm. The study was done in triplicate (n=3).

#### In vivo Pharmacodynamic Studies

*In vivo* studies were conducted under IAEC protocol approval no. IAEC/CCP/11/PR-07. The *in vivo* study using animals followed (inter)national ethical standards for the care and use of laboratory animals. For *in vivo* estimation of LDL (low density lipoproteins), HDL (high density lipoproteins), and triglycerides, the study was carried out in 36 healthy male rats weighing 200–250 g. The rats were randomly divided into six groups, containing six rats in each group. All the animals were fed a high fat diet (HFD) and water *ad libitum* for 11 weeks except the Control group (Group I). The HFD consisted of powdered normal pellet diet (NPD) (300 gm/kg), lard (275 gm/kg), casein (200 gm/kg), cholesterol (10 gm/kg), vitamin and mineral mix (60 gm/kg), dl-methionine (3 gm/kg), sodium chloride (2 gm/kg), and sucrose (150 gm/kg). The rats of Group I were fed NPD (commercial rat pellets from Kisan Feeds Ltd., Mumbai, India) for 11 weeks and water *ad libitum*. Group II served as the disease control group. The Standard group (Group III and IV) of rats received the pure drug, atorvastatin calcium (1 and 3 mg/kg/day, IP) and the Test group (Group V and VI) received a solid dispersion of atorvastatin (1 and 3 mg/kg/day, IP) for 3 weeks, started at the end of 8th week. Weekly body weights and daily food intakes were measured. At the end of the 11th week, blood samples were collected under ether anesthesia by retro-orbital puncture from overnight fasted rats. Serum was separated by centrifugation and was used to estimate serum total cholesterol, HDL (high density lipoproteins) cholesterol, and triglycerides [15]. Estimation of total cholesterol and HDL was done by using the Bayer Diagnostic kit (Bayer Diagnostic India Ltd) and the estimation of triglycerides was done by using Erba Diagnostics Manheim, Germany kit.

## Figures and Tables

**Fig. 4:**
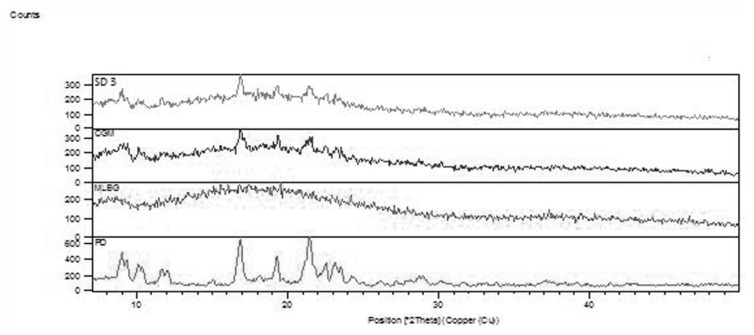
Overlay diagram of XRD of pure drug, MLBG, CGM, and SD3

**Tab. 1:** Characterization of LBG and MLBG

Parameters	LBG (Mean ± SD), n=3	MLBG (Mean ± SD), n=3
Swelling index (%)	284.62 ± 4.65	281.56 ± 2.25
Viscosity (cps)	1275 ± 40.28	418 ± 32.16
Hydration Capacity	2.38 ± 0.21	2.32 ± 0.18
Angle of Repose	36.58 ± 2.62	35.24 ± 2.34
Density (g/cc)		
Bulk Density	0.48 ± 0.12	0.50 ± 0.08
Tapped Density	0.62 ± 0.08	0.63 ± 0.06
CI (%)	22.58 ± 0.82	20.63 ± 0.68

**Tab. 2:** Evaluation of equilibrium solubility and content uniformity studies

Formulation	Equilibrium Solubility (μg/ml) (Mean ± SD), n=3	% Content Uniformity (Mean ± SD), n=3
Pure Drug	69.57 ± 0.56	–
SD 1	69.15 ± 0.63	92.6 ± 0.09
SD 2	79.03 ± 0.59	96.5 ± 0.06
SD 3	109.70 ± 0.48	100.1 ± 0.02
SD 4	109.49 ± 0.59	100.4 ± 0.01
SD 5	96.47 ± 0.65	100.3 ± 0.07
PM	70.00 ± 0.82	100 ± 0.08
CGM	108.44 ± 0.71	100.2 ± 0.05
KM	94.47 ± 0.64	99.8 ± 0.02
Marketed Product	–	100.98 ± 0.02

**Tab. 3:** Characteristic X-RD peaks of the pure drug, solid dispersion (SD3 batch), and Co-grinding mixtures

Pure drug		

Position (°2θ)	Relative intensity (%)	Area (cts*°2θ)
8.9716	54.25	67.90
10.0738	32.95	54.99
12.0574	28.79	24.03
16.8777	95.86	199.96
19.2928	59.25	98.88
21.4610	100.00	166.88
23.1231	46.33	57.99

**Solid Dispersion (SD3)**		

**Position (°2θ)**	**Relative intensity (%)**	Area (cts*°2θ)

9.1192	29.47	19.92
16.8950	100.00	33.80
19.3360	59.23	20.02
21.4785	50.34	34.03

**Co-grinding Mixture (CGM)**		

**Position (°2θ)**	**Relative intensity (%)**	Area (cts*°2θ)

9.1881	38.26	38.09
16.8505	100.00	49.78
19.384	88.25	26.36
21.3915	69.86	55.64
23.3291	41.42	24.74

**Tab. 4:** *In vivo* comparative evaluation of pure drug and SD3

Parameter (mg/dl)	Groups			

	Control	High fat diet (HFD)	Pure drug (Atorvastatin calcium)	Solid dispersion SD3
Total Cholesterol	100.00 ±13.10!	215.60 ± 22.04*	(a) 133.14 ± 12.07#! (b) 123.57± 9.25#!	(a) 125.80 ± 17.43#! (b) 94.59±6.40#!
LDL Cholesterol	38.55 ± 12.10!	138.09 ± 19.04*	(a) 61.21 ± 20.08#! (b) 54.59±12.35^#!^	(a) 56.09 ± 16.08#! (b) 36.55±6.24#!
HDL Cholesterol	51.32 ± 7.81!	34.53 ± 2.83*	(a) 49.59 ± 6.18#! (b) 45.23±5.59#!	(a) 55.43 ± 7.19#! (b) 58.45±4.52#!
Triglyceride	75.44 ± 18.10!	230.56 ± 14.51*	(a) 104.22 ± 18.14#! (b) 92±8.85#!	(a) 88.20 ± 14.21#! (b) 72.20± 4.21#!

(a) Indicates drug and SD group with 1 mg/kg/day and (b) indicates drug and SD group with 3 mg/kg/day.

* Indicates significant difference from the control group, at p < 0.05.

# Indicates significant difference from the HFD group, at p < 0.05.

! Indicates comparison difference from the control group and treatment group’s i.e pure drug and SD3 group.

**Tab. 5:** Composition of various solid dispersions

Formulation Code	Drug (mg)	Polymer (MLBG) (mg)
SD 1	100	200
SD 2	100	400
SD 3	100	600
SD 4	100	800
SD 5	100	1000
PM	100	600
CGM	100	600
KM	100	600
